# Loss of stem cell regenerative capacity within aged niches

**DOI:** 10.1111/j.1474-9726.2007.00286.x

**Published:** 2007-06

**Authors:** Morgan E Carlson, Irina M Conboy

**Affiliations:** Department of Bioengineering, University of California, Berkeley Berkeley, CA 94702, USA

**Keywords:** aged niche, aging, hESCs, myogenesis, regenerative potential, satellite cells

## Abstract

This work uncovers novel mechanisms of aging within stem cell niches that are evolutionarily conserved between mice and humans and affect both embryonic and adult stem cells. Specifically, we have examined the effects of aged muscle and systemic niches on key molecular identifiers of regenerative potential of human embryonic stem cells (hESCs) and post-natal muscle stem cells (satellite cells). Our results reveal that aged differentiated niches dominantly inhibit the expression of Oct4 in hESCs and Myf-5 in activated satellite cells, and reduce proliferation and myogenic differentiation of both embryonic and tissue-specific adult stem cells (ASCs). Therefore, despite their general neoorganogenesis potential, the ability of hESCs, and the more differentiated myogenic ASCs to contribute to tissue repair in the old will be greatly restricted due to the conserved inhibitory influence of aged differentiated niches. Significantly, this work establishes that hESC-derived factors enhance the regenerative potential of both young and, importantly, aged muscle stem cells *in vitro* and *in vivo*; thus, suggesting that the regenerative outcome of stem cell-based replacement therapies will be determined by a balance between negative influences of aged tissues on transplanted cells and positive effects of embryonic cells on the endogenous regenerative capacity. Comprehensively, this work points toward novel venues for *in situ* restoration of tissue repair in the old and identifies critical determinants of successful cell-replacement therapies for aged degenerating organs.

## Introduction

Embryonic stem cells (ESCs) are distinguished by their ability to self-renew and to differentiate into any other cell type via asymmetric cell divisions, in which one daughter cell maintains ‘stemness’ while the other daughter cell differentiates into a particular tissue type. ESCs, including those of human origin (hESCs), are derived from the blastocyst and can be propagated *in vitro* (Evans & Kaufman, 1981; Thomson *et al*., 1998; Wobus & Boheler, 2005). Their tremendous potential for organogenesis has created a great interest in using hESCs for replacing tissues and organs lost to disease, or old age (reviewed in Wobus & Boheler, 2005). As such, the use of hESCs is particularly important, due to the fact that adult organ stem cells are often limited in number, cell-fate plasticity, expansion capacity, telomere length, and lifespan (Mayhall *et al*., 2004). The general goal behind most cell-replacement approaches is to expand and then differentiate hESCs *in vitro*, thus producing a cell type of interest, such as neuronal, blood, endothelial, pancreatic, bone, and others. These differentiated cells are expected to replace their dysfunctional counterparts *in vivo*. The scope of disorders that can be potentially treated with a neoorganogenesis approach is large and includes many that are currently incurable, such as muscle atrophy, diabetes, Alzheimer's disease, Parkinson's disease, and other degenerative diseases that often accompany human aging (McDonald *et al*., 1999; Liu *et al*., 2000; Hori *et al*., 2002; Kim *et al*., 2002; Blyszczuk *et al*., 2003).

While many studies have focused on the derivation, propagation, and *in vitro* differentiation of hESCs (reviewed in Hoffman & Carpenter, 2005; Wobus & Boheler, 2005), relatively few have examined the properties of these cells and their more differentiated progeny in the aged, as opposed to the young, systemic and local organ environments. Recently published data suggest that these extrinsic cues become altered with age in ways that preclude activation of organ stem cells (such as satellite cells), inhibit repair-specific molecular signaling (such as delta-Notch), and interfere with productive tissue repair (Conboy *et al*., 2003, 2005; Janzen *et al*., 2006; Krishnamurthy *et al*., 2006; Molofsky *et al*., 2006). Furthermore, at least two lines of evidence suggest that stem cell-based tissue-replacement therapies might be hindered in the elderly, because all cells along the developmental lineage (e.g., stem cells, more differentiated progenitor cells or even tissues containing a pool of precursors) might rapidly ‘age’ and fail to contribute to organ repair when introduced into the old organism *in vivo*. First, in heterochronic tissue-transplantation studies, the age of the host environment determined the regenerative outcome, as both young and old skeletal muscle explants containing differentiated and precursor cells effectively regenerated in young, but not in old animals (Zacks & Sheff, 1982; Carlson & Faulkner, 1989). Second, using parabiotically paired young and old mice, the regenerative potential of muscle and liver was shown to be influenced by the age of the systemic environment (Conboy *et al*., 2005).

Thus, we sought to determine whether key molecular identifiers of stem cell properties, the rate of cell proliferation, and the myogenic capacity would be influenced by the age of extrinsic milieu, regardless of whether stem cells are embryonic or the more differentiated, muscle-specific satellite cells.

Satellite cells are muscle stem cells situated in direct contact with myofibers, the differentiated muscle cells. When myofibers are damaged, quiescent satellite cells are activated to proliferate and then differentiate into fusion-competent myoblasts that continue to proliferate and can form primary cultures, but are also capable of producing new, multinucleated myofibers or myotubes *in vitro* and *in vivo* (Morgan *et al*., 2002; Collins *et al*., 2005; Wagers & Conboy, 2005). Activated satellite cells express myogenic markers, such as Myf5, M-cadherin, and Paired box gene 7 (Pax7); fusion-competent myoblasts express high levels of desmin, and de novo generated myofibers or myotubes express embryonic myosin heavy chain (eMyHC) and continue to express desmin (Schultz & McCormick, 1994; Wagers & Conboy, 2005). While desmin can be also present in smooth and cardiac muscle cells, the isolation of hind limb skeletal muscle with subsequent purification of myofibers away from all interstitial cells, as well as purification of associated muscle stem cells results in primary cultures that are uniformly of skeletal muscle lineage. Every desmin^+^ cell in such cultures is a fusion-competent myoblast, and is able to produce multinucleated myotubes after 48 h of culture in differentiation-promoting medium [Dulbecco's modified Eagle's medium (DMEM) with 2% horse serum]. Some of these myogenic cells fuse into myotubes, even in the mitogen-rich medium [(Opti-MEM (Invitrogen, Carlsbad, CA, USA) with 5–10% mouse serum or DMEM with 10% fetal bovine serum, FBS] (Conboy & Rando, 2002; Conboy *et al*., 2003; and see below).

An experimental system was developed that (i) provided the ability to study the regenerative response of hESCs and of muscle stem cells in various heterochronic environments *in vitro*; and (ii) allowed examination of the effects of hESCs on muscle repair, *in vivo*, after transplantation into young vs. old hosts. This model allowed us to address both the negative effects of the aged niche on key stem cell properties and the positive effects of hESCs on the aged muscle-specific organ progenitor cells *in vitro*, and on the regenerative capacity of old muscle *in vivo*. The resulting data demonstrate that the composition of conserved extrinsic cues, regulating stem cell responses, becomes altered with age in ways that inhibit both hESCs and adult stem cell regenerative potential. Specifically, molecular markers of stem cell functionality, e.g. Oct4 (in hESCs) and Myf5 (in muscle stem cells), the rate of cell proliferation, and the capacity for myogenic differentiation are all dominantly inhibited by the aged systemic milieu, and by the old differentiated muscle tissue. However, while satellite cells are unable to deter the inhibitory affects of aged systemic and local niches, hESCs are capable of antagonizing the aged environments, thereby enhancing the regenerative potential of both young and old muscle stem cells *in vitro* and *in vivo*.

Thus, a complex interplay between negative regulation of hESCs and adult muscle stem cells by the aged niche, and positive regulation of the host's regenerative responses by hESCs will likely determine the success of hESC-based cell-replacement therapies in the old.

## Results

### Regenerative responses of adult muscle stem cells and hESCs are dominantly inhibited by the aged systemic milieu

**Fig. 1:**
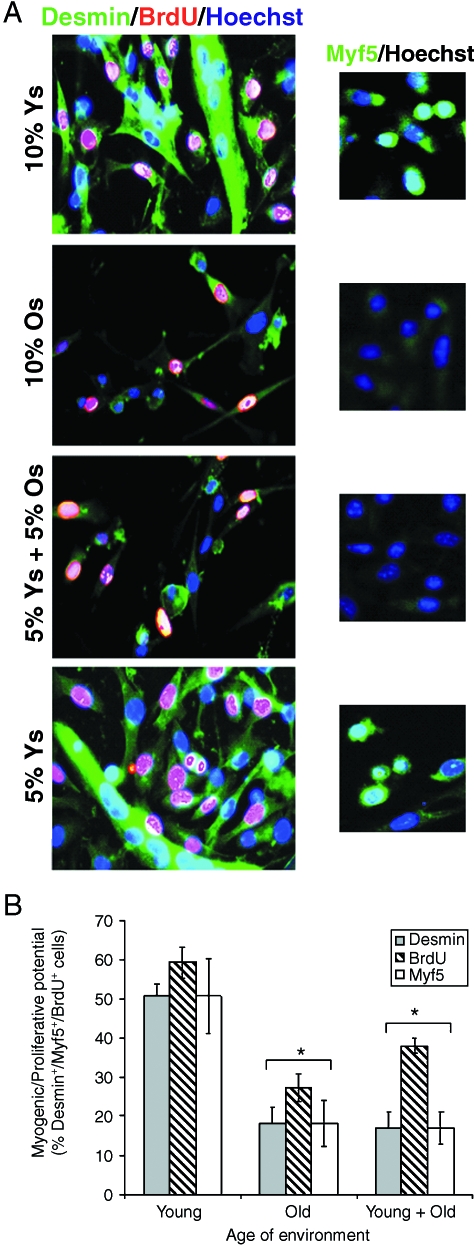
The age of sera determined the regenerative potential of satellite cells. (A) Young satellite cells were cultured either in 5% or 10% young (Young), 10% old (Old), or in a 5%+ 5% mouse sera combination (young + old). Cells were analyzed by immunofluorescence microscopy, using anti-BrdU (red), antidesmin (green) or anti-Myf5 antibodies (green, small panels). Similar results are shown for Pax7 immunodetection (Supplementary Fig. S1). Hoechst (blue) labeled nuclei. (B) Three independent experiments were quantified [300 young myofibers per experiment] as percentage of desmin^+^/Myf5^+^/BrdU^+^ de novo generated cells for each age and culture condition. On average, two to three fewer cells were generated when cultured in the presence of old. Shown are identical microscope fields at ×40 magnification. At least three independent experiments produced similar results. (*) indicates *P*≤ 0.001 as compared to young sera.

Previous work established that the upregulation of repair-specific molecular signaling mechanisms, such as Notch, and successful engagement of resident muscle stem cells in tissue repair are largely determined by the age of the systemic milieu, rather than by the cell-autonomous age of muscle cells, or by the differences in their numbers (Conboy & Rando, 2005; Conboy *et al*., 2005). Intriguingly, these experiments also hinted at a small but persistent inhibitory effect of the aged systemic milieu on the performance of young stem cells. Exploring this further, we found that young serum permits satellite cells to be myogenic, while old serum inhibits the satellite cell regenerative potential not only alone, but also when mixed with young serum, suggesting a dominant over-riding of ‘young’ serum factors (Fig. 1). Myofiber cultures, in which satellite cells have been activated by injury *in vivo*, were established from young (2–3 months) and old (22–24 months) C57-BL/6 male mice, as previously described (Conboy & Rando, 2002; Conboy *et al*., 2005). As previously shown, this method is well suited for the assessment of satellite cell regenerative myogenic capacity (Conboy & Rando, 2002; Wagers & Conboy, 2005). Isolated myofiber explants with associated satellite cells were cultured overnight in the presence of young or old serum (alone at 5% and 10%, and mixed at 5% young + 5% old); bromodeoxyuridine (BrdU) was added for the last 2 h of culture to measure the rate of cell proliferation. The effects of heterochronic systemic milieu on myogenic potential were examined as generation of proliferating myoblasts that express desmin and Myf5, and that spontaneously form multinucleated nascent myotubes. As shown in Fig. 1A and quantified in Fig. 1B, the age of sera clearly determined satellite cell regenerative potential and old serum strongly inhibited the myogenic potential of young satellite cells either when present alone, or when mixed with young sera. Similar data was obtained by using another myogenic marker, Pax7 (Supplementary Fig. S1). Additionally, there were two to three times fewer total cells generated in the presence of aged serum (not shown).

Importantly, it was not simply the dilution of young serum factors that resulted in diminished myogenic capacity when young and old sera were mixed, because young sera promoted robust myogenesis both at 10% and 5%. Thus, old serum factors dominantly inhibited the myogenic capacity of young satellite cells even in the presence of young serum. This observation suggests that satellite cells of young mice engage in efficient myogenic responses, in part, because the inhibitory influence of old circulatory milieu is absent.

These data reveal that the regenerative potential of young muscle stem cells is determined by the age of the systemic milieu, prompting us to investigate whether hESCs would similarly succumb to inhibitory factors present in the aged circulation.

**Fig. 2:**
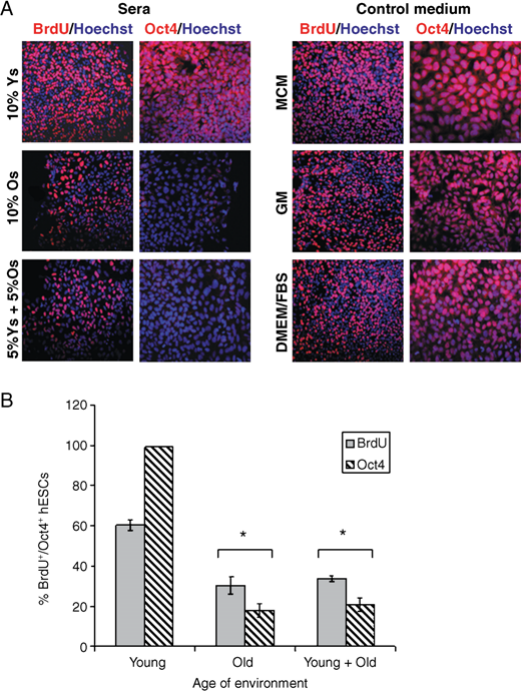
The regenerative potential of embryonic stem cells was negatively affected by aged mouse sera. (A) hESCs were cultured in MCM with 10% young (young) or old (old) mouse serum, or in three control media: *MCM* without mouse sera; *GM* (myoblast medium of Ham's F10 with 20% FBS) and *DMEM/FBS* (hESC differentiation medium of DMEM with 10% FBS). BrdU was added for the last 2 h of culture to measure the rate of cell proliferation. Immunodetection assays were performed for BrdU (red), Oct4 (red), and Ki67 (Supplementary Fig. S2). Hoechst (blue) labels nuclei. A high rate of hESC proliferation and Oct4 expression is displayed in all control media and in the presence of young mouse serum. In contrast, hESC proliferation and Oct4 expression are inhibited in the presence of old mouse serum, either alone or when mixed with young serum. MCM with mouse sera at 5% gave results similar to those observed with 10% young mouse sera or in control media (Supplementary Fig. S3). (B) Three independent experiments yielded similar results and were quantified as percentage of BrdU^+^ and Oct4^+^ cells for each culture condition. * indicates *P* < 0.001 as compared to young serum.

To determine the effects of aged serum on stem cell self-renewal/pluripotency, we analyzed hESC expression of Oct4 and studied the rate of hESC proliferation, by assessing BrdU incorporation (Fig. 2) and Ki67 expression (Supplementary Fig. S2). Specifically, these determinants of hESC regenerative potential were examined in the presence of heterochronic (young vs. old) mouse sera added to typical hESC medium, e.g., MEF-conditioned medium (MCM). Oct4 is expressed by self-renewing, pluripotent ESCs in culture, by the totipotent inner cell mass of the blastocyst and by the germ cells (Nichols *et al*., 1998; Pesce *et al*., 1999). Most cells in control cultures or young conditions expressed high levels of this marker of ‘stemness’, and maintained their normal phenotype and morphology throughout the various co-culture experiments performed in this study (see below).

Importantly, at 10% aged serum dramatically inhibited the self-renewal and proliferative potential of hESCs, as judged by highly diminished Oct4 expression and a lack of BrdU incorporation. Again, the inhibitory factors in the aged milieu were dominant over the young, as evidenced by a decline in Oct4 expression, the low rate of BrdU incorporation, and Ki67 expression in young and old mixed environments (5% young + 5% old sera in MCM). Similar to the data shown for adult stem cells (ASCs) (Fig. 1), it was not simply a dilution of young serum factors as hESCs robustly proliferated and expressed high levels of Oct4 when cultured with 5% young sera in MCM (Supplementary Fig. S3). Quantification of multiple independent experiments has demonstrated that hESC expression of Oct4 and BrdU incorporation have been reduced by two- to threefold in the aged milieu (Fig. 2B).

As expected, hESCs cultured in control media, including MCM alone that does not contain either young or old serum, also displayed a high rate of proliferation and Oct4 expression (Fig. 2, control medium). Additionally, in this experimental set-up there was no general inhibitory effect of sera *per se* on hESC proliferation and Oct4 expression, as 10% young mouse sera (young) and 10–20% of FBS (growth medium and DMEM/FBS) allowed for a high rate of cell proliferation and for uniformly high Oct4 levels (Fig. 2).

**Fig. 3:**
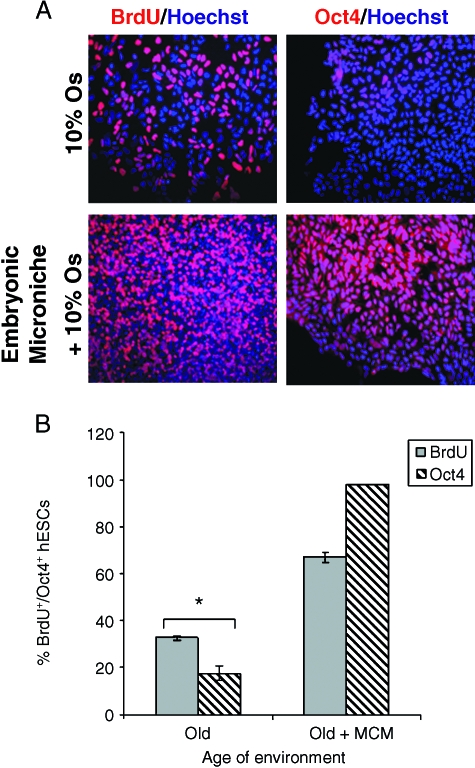
Embryonic stem cells produce youthful microniche in culture. (A) As opposed to immediate exposure to old mouse serum after passaging (10% old), preculturing of hESCs for 24 h in feeder-free conditions, e.g., Matrigel™ + MCM, prior to replacing MCM with MCM + 10% old mouse sera, resulted in continuously high BrdU incorporation and Oct4 expression (embryonic microniche + 10% old). BrdU was added for the last 2 h of culture to measure the rate of cell proliferation. Immunodetection of BrdU and Oct4 (both in red) was performed as described in Experimental procedures. Hoechst (blue) labels nuclei. (B) Three independent experiments yielded similar results and were quantified as percentage of BrdU^+^/Oct4^+^ for each condition. * indicates *P* < 0.001 as compared to ‘old + MCM’.

**Fig. 4:**
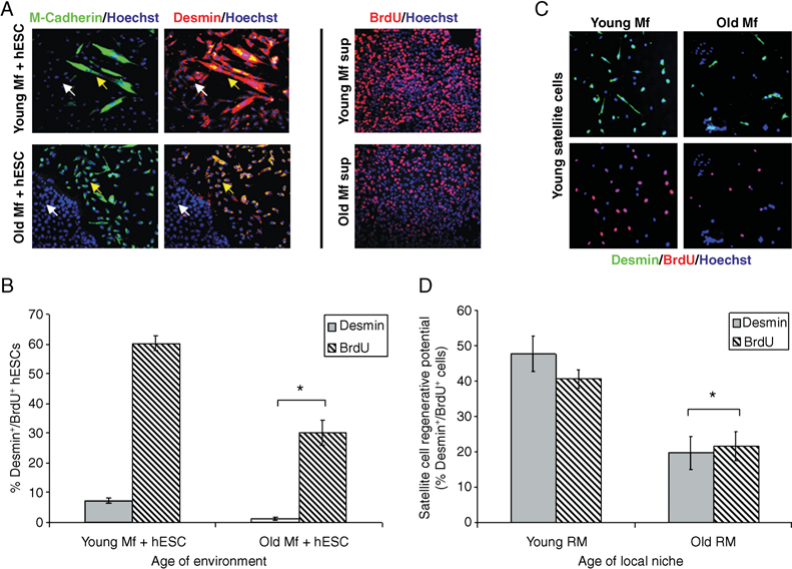
Aged muscle niche inhibits the regenerative potential of hESCs and satellite cells. (A) Immunodetection of a mouse-specific M-cadherin (green) or desmin (red; both human and mouse proteins are detected) revealed that hESCs underwent muscle lineage differentiation when co-cultured with young, but not old myofibers. The myogenic progeny of hESCs appears M-cadherin^−^/desmin^+^ (white arrow in young), as opposed to M-cadherin^−^/desmin^−^ hESCs that lack myogenic commitment (white arrow in old). M-cadherin^+^/desmin^+^ cells are the myogenic progeny of mouse satellite cells (yellow arrows). To assess the effects of secreted factors produced by young vs. old myofibers on the rate of hESC proliferation, transient, 2 h BrdU incorporation was examined in hESCs cultured for 48 h with supernatants produced by heterochronic myofiber explants (See Experimental procedures for details). As compared to young myofiber-derived supernatants (young myofiber supernant), exposure to old myofiber-derived supernatants (old myofiber supernant) inhibited hESCs proliferation, as judged by BrdU immunodetection (red). As expected, the rate of hESCs proliferation was high in control media (shown in Fig. 2). Hoechst (blue) labels nuclei in all experiments. Quantification of desmin^+^/BrdU^+^ hESCs in direct myofiber cocultures, or with muscle supernatants, is shown in (B). * indicates *P*≤ 0.001 as compared to young. (C) Transwell co-cultures between purified young satellite cells and myofibers isolated from uninjured young (young myofiber) and old (old myofiber) muscle demonstrated that satellite cell regenerative myogenic capacity was inhibited by the aged differentiated muscle. Myogenic potential was determined by the ability of satellite cells to generate proliferating desmin^+^ myoblasts (immunodetection shown in green) and by rate of proliferation (2 h BrdU incorporation; immunodetection shown in red). (D) Satellite cell regenerative potential was quantified as percentage of desmin^+^/BrdU^+^ cells for transwell co-cultures with young or old uninjured myofibers (i.e., RM, resting muscle). *n* = 3; * indicates *P*≤ 0.05 as compared to young.

When instead of immediate exposure to aged mouse serum, hESCs were first cultured overnight in MCM, these cells were no longer susceptible to the negative effects of old systemic milieu (Fig. 3), suggesting that hESC-produced factors established an embryonic microniche that may provide temporary protection from the aged environment. It appears that satellite cells do not have such anti-aging ability, because despite an initial activation in entirely young environments, e.g., after muscle injury to young muscle, isolated satellite cells remain susceptible to inhibition by the old mouse serum (Figs 1 and 4C). Similarly, culturing satellite cells isolated from noninjured muscle in growth-promoting medium for 1–2 days does not protect against the inhibitory affects of aged systemic milieu (not shown).

Comprehensively, these data establish that the inhibition of stem cell regenerative potential by the aged systemic milieu is conserved between species (mouse vs. human) and cell types (adult vs. embryonic stem cells). As summarized in Table 1, aged mouse sera similarly affected the expression of key molecular identifiers of both embryonic and adult stem cells, e.g., Oct4 in hESCs and Myf5 in mouse ASCs. As expected, adult mouse stem cells did not express Oct4, and hESCs did not express Myf5 in these experimental conditions (not shown). Moreover, aged systemic milieu had similar inhibitory effects on proliferation of hESCs and ASCs, suggesting that not only the regenerative capacity, but also the presence and expansion of stem cells will be significantly restricted in aged organs. Intriguingly, prolonged culturing of hESCs in their preferred *in vitro* conditions enables generation of an embryonic microniche that antagonizes the inhibitory influences of aged circulatory factors.

**Table 1:** Conservation of stem cell aging in the systemic environment

	Rate of proliferation ESC/ASC (percentage of BrdU)	Call-fate identifier ESC (percentage of Oct4)/ ASC (percentage of Myf5)
10% young	59.5 ± 0.8, 59.3 ± 4.0	99.0 ± 0.1, 50.7 ± 9.5
10% old	32.7 ± 2.1, 27.3 ± 3.5	17.6 ± 3.2, 18.1 ± 5.9
5% young + 5% old	31.0 ± 2.6, 38.0 ± 2.0	20.6 ± 3.5, 17.1 ± 4.2

Quantified results from Figs 1, 2 are summarized and presented as mean percentages from experimental replicates ± SE. Rate of proliferation (BrdU) and cell-fate identifier (Oct4 or Myf5) are shown for both ESCs and ASCs cultured in heterochronic systemic conditions of 10% young (young), 10% old (old) or in 5%+ 5% mouse sera combination (young + old). Results for 5% young mouse sera are very similar to those for 10% young mouse sera and are shown in Fig. 1 (ASCs) and Supplementary Fig. S3 (hESCs).

### The regenerative potential of hESCs and ASCs is inhibited by aged differentiated muscle

After establishing that the aged systemic niche negatively affects the regenerative capacity of hESCs and of ASCs, we then assessed whether myogenic potential and the rate of cell proliferation would be inhibited in hESCs and ASCs by the aged local muscle niche. Myofibers with associated satellite cells were isolated from young and old injured muscle, and were directly co-cultured with hESCs in typical hESC differentiation medium (DMEM/FBS). Similar to Fig. 1, the myogenic potential in these co-cultures was assayed by the expression of desmin, which is present in both fusion-competent myoblasts and newly formed myotubes. To analyze whether hESCs, mouse myogenic progenitor cells or both could express desmin in direct co-cultures, we costained these cells with a mouse-specific antibody to a myogenic marker, M-cadherin, which does not react with human protein, and a desmin-specific antibody that recognizes both mouse and human proteins. As shown in Fig. 4A, hESCs underwent myogenic differentiation in co-cultures with young myofibers (M-cadherin^−^/desmin^+^ mononucleated cells, white arrow in young). These myogenic progeny of hESCs in co-cultures with young myofibers could be of skeletal, smooth or cardiac muscle lineages (Debus *et al*., 1983; Fischman & Danto, 1985; Schultz & McCormick, 1994). As expected, the young mouse muscle progenitor cells (M-cadherin^+^/desmin^+^) were more advanced in their degree of myogenic differentiation, which was of skeletal muscle lineage, as judged by the formation of large, multinucleated de novo myotubes (yellow arrow in young). In addition to the myogenically differentiated human cells, co-cultures with young myofiber explants also contained some small undifferentiated hESC colonies, as determined by immunoreactivity to a human-specific antibody to the nuclear mitotic apparatus protein, NuMA and Oct4 expression (Supplementary Fig. S4).

**Fig. 5:**
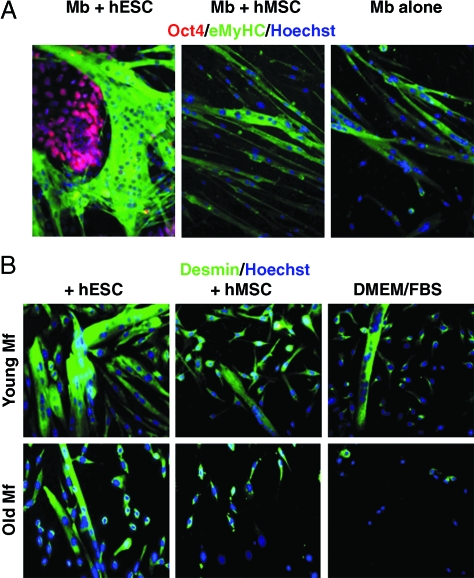
*In vitro* co-culture with hESCs enhanced myogenesis of mouse cells. (A) 1 × 10^5^ hESCs or control hMSCs were co-cultured with 5 × 10^6^ primary mouse myoblasts. hESCs expressing Oct4 (immunodetection shown in red) dramatically enhanced myotube formation of co-cultured mouse myoblasts (immunodetection of eMyHC is shown in green), as compared to co-cultures between mouse myoblasts and human mesenchymal stem cells (Mb + hMSCs) or myoblasts alone (Mb alone). Experiments were carried out in myoblast differentiation medium. Hoechst (blue) labels nuclei throughout this figure. (B) 1 × 10^5^ hESCs or control hMSCs were co-cultured with young or old myofiber-associated satellite cells, as described in Experimental procedures. Co-culture with hESCs (myofiber + hESC), but not hMSCs (myofiber + hMSC) or control medium (DMEM/FBS), greatly enhanced the myogenic potential of both young and old myofiber-associated satellite cells, based on immunodetection of percentage of desmin^+^ de novo generated myoblasts and multinucleated myotubes. These experiments were carried out in GM. Shown are myogenic responses of mouse cells only, judged by lack of immunoreactivity to human-specific/hESC-specific antigens, such as NuMA and Oct4; and presence of mouse-specific immunoreactivity, e.g., M-cadherin (not shown). Both young and old myofiber associated satellite cells exhibited considerable myogenic improvement over control conditions. *n* = 3.

In contrast, when co-cultured with the aged mouse myofibers, only mouse cells appeared desmin^+^ (Fig. 4A, yellow arrow in old). These aged myogenic cells were of skeletal muscle lineage, based on spontaneous generation of multinucleated myotubes (see Fig. 5B) and based on induced differentiation into myotubes in DMEM + 2% horse serum (not shown). Importantly, the myogenic differentiation of hESCs failed in the aged co-cultures (Fig. 4A, white arrow in old). Furthermore, colonies of hESCs in co-cultures with aged myofibers typically differentiated into cells with fibroblast morphology, which lacked Oct4 expression (not shown). Spontaneous production of desmin^+^ myogenic cells in control hESC cultures without myofibers, or with young/old mouse sera was less than 0.1% (not shown).

In concert with the conservation of inhibitory affects of aged systemic niche, the negative influence of local muscle niche was also found to be conserved in its inhibition of hESC and ASC regenerative responses. Specifically, the myogenic capacity (generation of desmin^+^ myoblasts) was inhibited in young satellite cells co-cultured in a transwell system with aged myofibers (Fig. 4B). In addition, hESC and ASC proliferation (BrdU incorporation) was also inhibited by aged differentiated muscle (Fig. 4A,C). These data suggest that not only systemic but also local organ niches would inhibit key stem cell properties, e.g., myogenic capacity and the rate of proliferation in the aged organism. The conserved inhibitory influences of the differentiated muscle niche on hESC and ASC regenerative responses are summarized in Table 2.

**Table 2:** Conservation of stem cell aging in the local organ niche

	Rate of proliferation ESC/ASC (percentage of BrdU)	Myogenic differentiation ESC/ASC (percentage of desmin)
Young myofiber	60.2 ± 2.5, 40.5 ± 2.6	7.4 ± 0.9, 47.6 ± 5.0
Old myofiber	30.1 ± 4.3, 21.5 ± 4.1	1.3 ± 0.7, 19.7 ± 4.7

Quantified results from Fig. 4 are summarized and presented as mean percentages from experimental replicates ± SE. Rate of proliferation (BrdU) and myogenic differentiation (desmin) are shown for both ESCs and ASCs, in the presence of young vs. old differentiated muscle environments (young myofiber or old myofiber).

### hESCs indirectly enhance and rejuvenate the regeneration of skeletal muscle

While hESC properties were inhibited by aged differentiated muscle, the myogenic potential of aged satellite cells seemed to be enhanced by co-cultures with hESCs (Fig. 4A). Therefore, we further explored the enhancing and rejuvenating effects of hESCs on myogenic potential *in vitro* and *in vivo*, using human mesenchymal stem cells (hMSCs) as a negative control. First, we examined the effects of hESCs on myotube generation by co-culture with primary myoblasts freshly derived from activated-by-injury satellite cells (Conboy *et al*., 2003). As shown in Fig. 5A (Mb + hESC), primary myoblasts underwent very rapid and robust nascent myotube formation, when co-cultured with hESCs for 48 h in myoblast differentiation medium. Namely, remarkably large fused myotubes containing approximately 50–70 nuclei formed around hESCs colonies (Fig. 5A). In contrast, when co-cultured with hMSCs, myotube formation was no greater than in myoblast cultures alone (Fig. 5A, Mb + hMSC and Mb alone). Encouraged by these data, we analyzed the myogenic potential of young and old satellite cells co-cultured with hESCs for 48 h. As shown in Fig. 5B, hESCs conferred a much-enhanced myogenic capacity on both young and, importantly, old myofiber-associated satellite cells (rapid formation of desmin^+^ myogenic cells, many of which formed de novo multinucleated myotubes). Control co-cultures of these satellite cells with hMSCs displayed no enhanced myogenicity. In summary, while the myogenic potential (production of desmin^+^ fusion-competent cells) was more pronounced in young vs. old myofiber-associated satellite cells under all experimental conditions, a finding that is consistent with previous data (Conboy *et al*., 2003), a clear increase in myogenic potential of old satellite cells was noted in co-cultures with hESCs, as compared to control cultures devoid of hESCs (Fig. 4A,B).

Interestingly, in addition to the rejuvenating effects of direct co-cultures shown in Fig. 5, soluble factors present in hESC-conditioned culture supernatants were also able to enhance myogenesis of aged satellite cells (Supplementary Fig. S5). Thus, in agreement with the notion that an established embryonic microniche antagonizes the inhibitory effects of the aged environment on stem cell responses (Fig. 3), the hESC-produced factors enhanced myogenic capacity of even old mouse satellite cells.

**Fig. 6:**
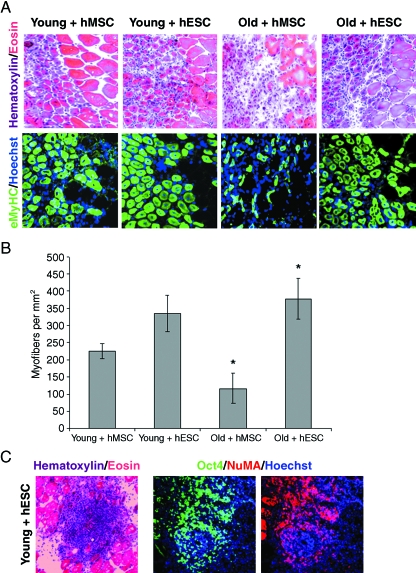
Skeletal muscle regeneration following hESC transplantation is a balance between the inhibitory influence of aged niches and the rejuvenating effects of hESCs. Young and old tibialis anterior and gastrocnemius muscles were injured by cardiotoxin injection. hESCs or hMSCs were transplanted at the site of injury and were analyzed by cryosectioning at Day 5 after injury (as described in Experimental procedures). (A) Newly regenerated myofibers were detected using eMyHC-specific antibody (green) and staining with H&E. In H&E staining, newly regenerated areas contain smaller, immature myofibers with centrally located nuclei. Uninjured myofibers are much larger, by comparison, with peripherally restricted nuclei. Poorly regenerated areas lack new myofibers and contain areas of fibrosis and inflammation. eMyHC immunodetection is specific for regenerating areas of muscle only. Both assays showed dramatic enhancement of muscle regeneration in ‘old + hESC’ vs. ‘old + hMSC’. Regeneration improvement was also seen in young + hESC, as compared to young + hMSC. (B) Quantification of muscle regeneration was performed by analyzing the density of newly formed myofibers per mm^2^ of injury site, which is the volume that typically covers the whole injured area. Multiple, 10 µm H&E sections were examined through the entire volume of injury in multiple, independently injured muscles. *n* = 20; * indicates *P* < 0.001 (‘old + hMSC’ compared to young + hMSC and ‘old + hMSC’ compared to ‘old + hESC’. (C) H&E and immunofluoresence staining for Oct4, and a human-specific antibody to NuMA, revealed the failure of hESCs to expand or persist in old, but the presence of hESCs in young muscle at 5 days post-transplantation. Hoechst (blue) labels nuclei.

Establishing that hESC-produced factors enhance adult myogenesis and rejuvenate the regenerative capacity of even aged satellite cells *in vitro* prompted us to examine whether the regeneration of old injured muscle will be improved by hESC transplantation *in vivo*. Additionally, based on the data shown above, we speculated that even if the host's repair capacity is improved, hESCs themselves will not be efficiently maintained or expanded in the context of old systemic and local organ environments, and will not directly contribute to the repair of aged skeletal muscle. To test these hypotheses, we injected 5 × 10^5^ hESCs or control hMSCs into the tibialis anterior (TA) and gastrocnemius muscles of young and old mice at 24 h after cardiotoxin-induced injury, when activation/proliferation of endogenous satellite cells normally begins (Conboy *et al*., 2003, 2005; Wagers & Conboy, 2005). To avoid immune response against hESC antigens, mice were immunosuppressed using FK506 (Ito & Tanaka, 1997; Dumont, 2000). Muscle was isolated 5 days post-injury, when nascent differentiated myofibers normally replace the damaged tissue (Conboy *et al*., 2003), and 10 µm cryosections were analyzed for the success in tissue repair using hematoxylin and eosin (H&E) histochemistry and eMyHC immunodetection. H&E analysis reveals newly formed myofibers, based on their smaller size and centrally located nuclei. Additionally, de novo myofibers in the damaged area appear positive for eMyHC, while undamaged myofibers remain negative. As shown in Fig. 6A and quantified in 6B, injection of hESCs significantly enhanced regeneration of skeletal muscle. Remarkably, this positive embryonic effect was especially pronounced in old tissue.

Importantly, such enhanced and rejuvenated muscle repair stems from an indirect induction, as hESCs themselves (or control hMSCs) did not physically contribute to the mouse myofibers, as judged by near absence (less than 0.1%) of human-specific NuMA^+^ nuclei in de novo desmin^+^ myofibers, analyzed through multiple injury sites. An example of one regenerated myofiber from young muscle injected with hESCs, with NuMA^+^ nucleus in a field of NuMA^−^/desmin^+^ mouse myofibers, is shown in Supplementary Fig. S6. No such NuMA^+^/desmin^+^ myofibers were detected in aged regenerated muscle (not shown).

In agreement with the *in vitro* data, establishing that aged systemic and local niches inhibit hESC proliferation and Oct4 expression (Figs 2 and 4 and Supplementary Fig. S2), hESCs failed to expand or even persist in old muscle, as judged by the absence of NuMA^+^/Oct4^+^ hESC-derived cells in the aged tissue. In contrast, colonies of Numa^+^/Oct4^+^ hESC-derived cells that did not undergo myogenic differentiation were easily detected in young regenerating muscle (Fig. 6C). This finding validates several technical aspects of these experiments, and confirms the contrasting effects of young and old systemic and local organ niches on hESC self-renewal.

These data further confirm and extrapolate our findings and demonstrate that when exposed to both aged systemic and local organ niches, hESCs fail to persist and do not contribute to tissue repair directly. At the same time, these embryonic cells indirectly but significantly improve the repair of aged injured muscle *in vivo*.

## Discussion

The data presented here establish for the first time that both the local environment of old differentiated organ, e.g., skeletal muscle and the systemic milieu dramatically affect the regenerative potential of both hESCs and mouse post-natal myogenic progenitor cells. Not only are the factors promoting myogenic differentiation and proliferation of hESCs likely to become depleted with age, but the aged systemic and local organ niches are likely to contain dominant inhibitors of ASC and hESC regenerative potential (Figs 1, 2, and 4, summarized in Tables 1 and 2). Importantly, the similar inhibitory effects of old mouse serum and old myofibers on satellite cell (Figs 1 and 4C) and hESC (Figs 2 and 4A) proliferation and regenerative capacity suggest the conservation of elements in age-specific extrinsic regulatory mechanisms between evolutionarily distinct species and stem cell types. Additionally, a similarity in the inhibitory properties between systemic and local organ niches is also of interest and may indicate that molecules produced by old tissues have circulatory/endocrine activity; and/or that age-specific systemic inhibitory components become deposited in the old tissues.

Humans display broad genetic polymorphisms and behavioral variations, which makes the identification of age-specific molecular changes complicated. In contrast, laboratory mice are genetically and environmentally controlled. Establishing that age-specific signals, regulating stem cell responses, are evolutionarily conserved and soluble enables the formation of rational approaches for the identification and characterization of the inhibitors involved, and for revealing the precise timing of their first appearance in serum and differentiated tissues with advancing age.

Significantly, these experiments have also revealed that not only are hESCs able to protect themselves against the negative influences of aged mouse sera (Fig. 3), but these cells also produce factors that dramatically enhance the myogenic capacity of primary myoblasts and young and old satellite cells (Fig. 5), and also significantly improve repair of young and old injured muscle *in vivo* (Fig. 6). Identification of these embryonic factors would allow us to potentially enrich the arsenal of therapeutic tools for combating age-specific degenerative disorders.

The interactions between hESCs and heterochronic differentiated niches, initially identified *in vitro*, have been confirmed by *in vivo* experiments. Namely, while the regenerative capacity, or presence, of hESCs is greatly restricted in aged, as compared to young skeletal muscle (where transplanted cells experience both old systemic and local environments), embryonic cells indirectly enhance and rejuvenate muscle repair when introduced at the time of muscle stem cell activation in the host, e.g., at Day 1 after the injury (Fig. 6). It remains to be determined whether the percentage of hESCs direct contribution to desmin^+^ myofibers in young muscle will be increased by transplanting these cells at a different time-point after muscle injury, e.g., at Days 3–5 (as in co-cultures with myofibers pre-injured for 3 days, Fig. 4A). In any case, the virtual lack of hESC and hMSC direct contribution to the newly regenerated skeletal muscle, when small numbers of these cells were injected into injured tissue, is completely consistent with the body of previous data demonstrating that myofiber-associated satellite cells conduct rapid and robust muscle repair and greatly outnumber injected human cells (Collins *et al*., 2005; Wagers & Conboy, 2005); that compared to muscle-specific satellite cells, the myogenic differentiation of hESCs *in vitro* remains very small (Fig. 5, Table 2), and that control hMSCs are not normally myogenic unless these cells overexpress exogenous constitutively active domain of Notch (Dezawa *et al*., 2005).

Intriguingly, the failure of hESCs to strive in old skeletal muscle might represent a therapeutically desirable outcome. For example, while in young tissue hESC derivatives putatively would go on to produce teratomas, it is unlikely that teratoma formation would occur after hESC transplantation into aged skeletal muscle. Thus, the indirect beneficial effects of hESCs on tissue repair are unlikely to be compromised by the oncogenic properties of these embryonic cells in the context of old skeletal muscle.

Comprehensively, the results of this work increase our understanding of aging as a process, reveal evolutionary conserved age-specific interactions between stem cells and their differentiated niches, and suggest novel therapeutic approaches for improving the regenerative responses of endogenous or transplanted stem cells in old individuals.

## Experimental procedures

### Animal strains

Young (2–3 months), C57-BL/6 male mice were obtained from pathogen-free breeding colonies at Jackson Laboratories (Bar Harbor, ME, USA). Aged 22–24 months C57-BL/6 male mice were obtained from the National Institute on Aging (NIH). Animals were maintained in the North-West Animal Facility of the University of California, Berkeley, CA, USA, and handled in accordance with the Administrative Panel on Laboratory Animal Care at UC Berkeley.

### Muscle injury and isolation

Myofiber cultures, in which satellite cells were activated by *in vivo* injury, were set up as previously described (Conboy & Rando, 2002; Conboy *et al*., 2005). Briefly, mice were injured by direct injection with 5 ng cardiotoxin (CTX-1) (Sigma, St Louis, MO, USA) into the tibialis anterior and gastrocnemius muscles using a 28-gauge needle. After 1–5 days post-injection, injured or uninjured muscle tissue was dissected out. Once isolated, whole muscle was prepared for cryosectioning (see below) or myofiber fragments were obtained from hind limb muscles by enzymatic digestion (see below), trituration, and multiple sedimentation and washing procedures. Additionally, blood was collected from mice for the isolation of sera. Briefly, blood cells were coagulated at 37 °C for 15’ and then were centrifuged repeatedly at 5900 *g*, 4 °C in a microfuge for 3’ to isolate sera. Mixtures of young and old sera were made 1 : 1. For example, in 5%+ 5% conditions, 50 µL of young and 50 µL old serum were added to 900 µL of culture medium (Opti-MEM or MCM, see co-culture procedures below).

### Myofiber explant cultures

Explant and primary cell cultures were generated from C57-BL/6 mice, as previously described (Conboy & Rando, 2002; Conboy *et al*., 2003). Dissected gastrocnemius and tibialis anterior muscles underwent enzymatic digestion at 37 °C in DMEM (Invitrogen)/Pen-Strep (Invitrogen)/0.2% Collagenase Type IIA (Sigma) solution. Isolated fibers were resuspended in GM (Ham's F10 nutrient mixture (Mediatech, Inc., Herndon, VA, USA), 20% FBS (Mediatech), 5 ng mL^−1^ bFGF (Chemicon, Temecula, CA, USA) and 1% Pen-Strep, and cultured on ECM-coated (BD Biosciences, San Jose, CA, USA) plates (diluted 1 : 500 in PBS). Cultures of primary myoblasts were derived from isolated fibers, through repeated passaging, and were maintained in GM. Myoblast differentiation medium [DMEM, supplemented with 2% horse serum (Mediatech)] was used to promote rapid formation of myotubes from cultured myoblasts (Morgan & Partridge, 2003).

### Human embryonic and mesenchymal stem cell culture

The federally approved hESC line, H7 (NIH no. WA07, obtained from WiCell Research Institue, Madison, WI, USA), was used in accordance with the UC Berkeley and UC San Francisco Committee on Human Research guidelines, and in accordance with NIH guidelines. To propagate hESCs, routine culturing and maintenance was performed using standard *in vitro* conditions for both feeder-dependent and feeder-free cultures (Geron Corporation, 2002). Briefly, hESCs grown on MEFs were cultured in standard hESC medium [Knockout™ DMEM, 20% KSR, 1% NEAA, 1 mm l-glutamine (Invitrogen), 0.1 mmβ-mercaptoethanol (Sigma)] and were supplemented with 4 ng mL^−1^ hbFGF (Invitrogen). Feeder-free hESC cultures were maintained in MEF-conditioned hESC medium (MCM), 4 ng mL^−1^ hbFGF. Differentiation medium for hESCs (DMEM/FBS) was made by replacing KSR with 20% FBS (Hyclone, Logan, UH, USA). hMSCs were maintained in mesenchymal stem cell GM, MSC-GM™ and were cultured according to supplier recommendations (Cambrex Walkersville, MD, USA). hESCs and hMSCs were typically seeded onto chambered slides coated with a 3% GFR Matrigel™ (BD Biosciences) substrate in PBS. Cells were typically incubated for 48 h at 37 °C, 5% CO_2_, under the various experimental conditions employed, then were fixed with 70% EtOH/PBS at 4 °C. hESCs and hMSCs were analyzed 24–48 h after experimental treatments, during which no apoptosis-related differences in cell numbers were observed.

### Heterochronic co-culture systems

*Heterochronic systemic cultures* were established by culturing myofiber explants (in GM) or hESCs (in MCM) in the presence of young, old or young + old sera for 48 h (Figs 1 and 2 and Supplementary Figs S1–3). In such cultures, hESCs were passaged immediately prior to sera exposure. In contrast, preculturing of hESCs for 24 h in MCM, prior to replacing MCM with MCM + 10% old mouse sera was done for embryonic microniche experiments (Fig. 3). For heterochronic local organ niche cultures, hESCs were co-cultured directly with myofiber explants for 48 h in GM, or were cultured in the presence of supernatants derived from cultured myofiber explants for 48 h (Figs 4A and 5). Specifically, 1 × 10^5^ hESCs or control hMSCs were co-cultured with identical volume, e.g., 100 µL, of young or old myofiber fragments with their associated satellite cells (Fig. 5). In experiments shown in Supplementary Fig. S5, culture-conditioned supernatant produced by hESCs grown in MCM was used as a medium in which 1 × 10^5^ of myofiber-associated young or old satellite cells were cultured for 48 h. In direct co-cultures, mouse vs. human cells were distinguished by immunodetection with human-specific/hESC-specific and mouse-specific antibodies (Supplementary Fig. S4 and see below). To prepare muscle supernatants, explants were cultured for 24 h in GM and cellular debris was removed from conditioned media by multiple rounds of centrifugation. The absence of cells was confirmed by microscopic examination. To mimic the local organ niche for satellite cell assays (Fig. 4B), 1.0 µm transwell (Corning, NY, USA) co-cultures of uninjured explants with activated satellite cells were established. Activated-by-injury (24 h post-injury) satellite cells were seeded onto ECM-coated 12-well plates in Opti-MEM (Invitrogen) and 5% FBS. Transwells were placed over satellite cells and contained isolated myofiber explants from uninjured young or old muscle (i.e., resting muscle). Satellite cells were cultured for 72–96 h in the presence of myofiber explants and were fixed for immunodetection, as described above.

### Cell transplantation

hESCs were grown on MEFs and expanded in 6-well plates. Cells were treated with 1 mg mL^−1^ Collagenase Type IV (Invitrogen) for 5–10 min, were washed and then incubated with 0.5 mg mL^−1^ Dispase (Invitrogen) to lift only human cell colonies. Isolated hESCs were washed several times and resuspended in 100 µL hESC medium. Similarly, hMSCs were expanded in 6-well plates, lifted with Trypsin/EDTA (Invitrogen), washed and resuspended in 100 µL hESC medium. Approximately 5 × 10^5^ hESCs or hMSCs were injected into 24 h post-injured gastrocnemius and tibialis anterior muscles of young and old mice, using a 21-gauge needle. Immunosuppression of animals was achieved by intraperitoneal injection of 1 mg kg^−1^ FK506 (Sigma) at 48 h prior to cell transplantation, and on each day following transplantation.

### Immunodetection and histological analysis

To assay the affects of heterochronic local and systemic environments on stem cell regenerative potential, hESC, hMSC, and myofiber-derived precursor cell cultures were fixed with 70% EtOH/PBS at 4 °C, and were analyzed by indirect immunofluorescence. Combinations of antibodies were used to co-stain cultures and histosections, in order to determine the percentages of cells that proliferated or differentiated and to distinguish hESCs from mouse cells. Antibodies to the myogenic transcription factors, Myf5/Pax7, the intermediate filament protein, desmin, and the marker of newly formed myotubes, eMyHC, were used to reveal commitment to myogenic differentiation. Cell commitment to this differentiation program was assessed by the efficiency of myotube formation, estimated by the number of nuclei per myotube. Ki67, a cell cycle related nuclear protein consistently absent in quiescent cells, was used as a marker for proliferation. Whereas Ki67 appears in all active phases of the cell cycle, BrdU staining allowed exclusive detection of cells in S-phase, thereby enabling accurate quantification of DNA synthesis. In select cultures, 10 µm BrdU was added for 2 h prior to fixation. BrdU-specific immunostaining required nuclear permeabilization with treatment of 4N HCl. hESCs were distinguished from mouse cells by using a species-specific antibody to the cell-surface marker M-cadherin for murine and the nuclear marker NuMA for human cells. Antibodies to Oct4 were used as a marker of hESC self-renewal/pluripotency. Following permeabilization in PBS, +1% FBS, +0.25% Triton X-100, cells were incubated with primary antibodies (concentration determined as per manufacturer's recommendations) for 1 h at room temperature in PBS, +1% FBS, washed several times, and then incubated with fluorophore-conjugated, species-specific secondary antibodies (diluted 1 : 500 in PBS + 1% FBS) for 1 h at room temperature. For histological analysis, dissected muscle was treated in a 25% sucrose/PBS solution, frozen in OCT compound (Tissue Tek) and cryosectioned at 10 µm. Immunostaining was performed in the manner described above, or H&E staining of cryosections was performed. Nuclei were visualized by Hoechst staining for all immunostains. Samples were analyzed at room temperature by using a Zeiss Axioscope 40 fluorescent microscope, and imaged with an Axiocan MRc camera and AxioVision software. All images depict identical microscope fields at ×20 magnification, unless otherwise noted.

### Reagents

Antibodies to Oct4 (ab18976), BrdU (BU1/75 (ICR1), and Ki67 (ab15580) were purchased from Abcam (Cambridge, MA, USA). Antibody to M-cadherin (clone 12G4) was acquired from Upstate Biotechnology (Lake Placid, NY, USA), and NuMA antibody (Catalog number NA09L) from EMD Biosciences (San Diego, CA, USA). Antibody to developmental eMyHC (clone RNMy2/9D2) was acquired from Vector Laboratories (Burlingame, CA, USA). Myf5 (GTX77876) and Pax7 (GTX77888) antibodies were obtained from GeneTex (San Antonio, TX, USA). Desmin antibodies (clone DE-U-10 and Catalog number D8281), BrdU labeling reagent and FK506 (Catalog number F4679) were obtained from Sigma. Fluorophore-conjugated secondary antibodies (Alexa Fluor) were obtained from Molecular Probes (Eugene, OR, USA).

### Statistical analyses

A minimum of three replicates were undertaken for each experimental condition. Quantified data are presented as means ± SE. Significance testing was performed using one-way analysis of variance (anova) to compare data from different experimental groups. *P* values of < 0.05 were considered as statistically significant.

## Supplementary material

The following supplementary material is available for this article:

Fig S1. The expression of Pax7 was reduced in satellite cells cultured in the presence of aged sera. Young satellite cells were cultured in 10% young (young) or 10% old (old). Immunodetection was performed using Pax7 antibody (green). Hoechst (blue) labels nuclei. High numbers of Pax7^+^ myogenic cells were detected in the presence of 0% young, but not in the presence of 10% old. Shown are identical microscope fields at ×40 magnification. At least three independent experiments produced similar results. Fig S2. Aged mouse serum negatively affected the regenerative potential of hESCs. i67 expression (immunodetection shown in red) by hESCs cultured in the presence of 10% oung, old or 5% young + 5% old mouse sera or control medium (DMEM/FBS or GM) revealed high rate of hESC proliferation in young mouse serum, and an inhibition of hESC roliferation in old or in young + old combinations. Hoechst (blue) labels nuclei. hree ndependent experiments yielded similar results and were quantified, as percentage of Ki67^+^ cells for each culture condition (*P* > 0.001 as compared to young). Fig S3. hESCs cultured with 5% young mouse serum robustly proliferated and xpressed Oct4. Immunodetection of BrdU incorporation (green) and Oct4 expression (red) y hESCs was robust in cultures with 5% young mouse serum (5% young) in MCM medium. oechst (blue) labels nuclei. At least three independent experiments produced similar results. Fig S4. Small colonies of undifferentiated hESCs persisted in co-cultures with ouse myofibers. Nuclear Oct4 (immunodetection is shown in red), co-localizes with a uman-specific nuclear mitotic apparatus protein (NuMA immunodetection is shown in reen). Hoechst (blue) labels nuclei. hESCs are shown as Oct4^+^/NuMA^+^. Mouse cells ppear only as Hoechst stained. Fig S5. The regenerative potential of aged satellite cells is improved by ecreted, soluble hESC-derived factors. Immunodetection with an antidesmin antibody (green) hows that myogenic capacity (generation of desmin^+^ de novo myoblasts and myotubes) is high in young myofiber-associated satellite cells, and is rejuvenated in old myofiber-associated satellite cells cultured in hESC-conditioned supernatants Hoechst (blue) labels nuclei. Three independent experiments yielded similar results. Fig S6. The majority of injured myofibers were regenerated by endogenous mouse cells, and not by hESCs *in vivo*. Observed enhancement of muscle repair is not from direct contribution of hESCs to newly formed myofibers, as judged by near absence (> 0.1%) of human-specific NuMA^+^ nuclei in de novo desmin^+^ myofibers (desmin immunodetection is shown in green). White arrow depicts a single regenerating myofiber from young muscle injected with hESCs, with NuMA^+^ nucleus (immunodetection is shown in red) in a field of NuMA^−^/desmin^+^ mouse myofibers. NuMA^+^/desmin^+^ myofibers were not detected through injury sites in aged regenerated muscle. Multiple, 10 μM NuMA/desmin sections were examined through the entire volume of injury from independently injured muscles. Myofibers were examined per total area of injury. n = 20.

This material is available as part of the online article from http://www.blackwell-synergy.com/doi/abs/10.1111/j.1474-9726.2007.00286.x

(This link will take you to the article abstract).
